# Evidence of direct complementary interactions between messenger RNAs and their cognate proteins

**DOI:** 10.1093/nar/gkt618

**Published:** 2013-07-18

**Authors:** Anton A. Polyansky, Bojan Zagrovic

**Affiliations:** Department of Structural and Computational Biology, Max F. Perutz Laboratories, University of Vienna, Campus Vienna Biocenter 5, A-1030 Vienna, Austria

## Abstract

Recently, the ability to interact with messenger RNA (mRNA) has been reported for a number of known RNA-binding proteins, but surprisingly also for different proteins without recognizable RNA binding domains including several transcription factors and metabolic enzymes. Moreover, direct binding to cognate mRNAs has been detected for multiple proteins, thus creating a strong impetus to search for functional significance and basic physico-chemical principles behind such interactions. Here, we derive interaction preferences between amino acids and RNA bases by analyzing binding interfaces in the known 3D structures of protein–RNA complexes. By applying this tool to human proteome, we reveal statistically significant matching between the composition of mRNA sequences and base-binding preferences of protein sequences they code for. For example, purine density profiles of mRNA sequences mirror guanine affinity profiles of cognate protein sequences with quantitative accuracy (median Pearson correlation coefficient *R* = −0.80 across the entire human proteome). Notably, statistically significant anti-matching is seen only in the case of adenine. Our results provide strong evidence for the stereo-chemical foundation of the genetic code and suggest that mRNAs and cognate proteins may in general be directly complementary to each other and associate, especially if unstructured.

## INTRODUCTION

In the 50 years since the discovery of messenger RNA (mRNA) (1), the relationship between this key biopolymer and proteins has been studied predominantly in the context of transmission of genetic information and protein synthesis. Recently, however, evidence of direct non-covalent binding between mRNAs and a number of functionally diverse proteins has been provided, including surprisingly various metabolic enzymes, transcription factors and scaffolding proteins with hitherto uncharacterized RNA-binding domains (2–5). It has been found that such mRNA–protein complexes frequently participate in the formation of RNA droplets in the cell (e.g. P-bodies), which display all features of a separate cytoplasmic microphase and open up new paradigms in cell biophysics (6–8). What is more, several proteins have been found over the years to directly bind their own cognate mRNAs, including among others thymidylate synthase, dihydrofolate reductase and p53 (2,9–14), with binding sites in both translated and untranslated mRNA regions. The functional significance of such cognate interactions has been clearly ascertained in some cases [e.g. translational feedback control (12)], but it is far from clear how general and functionally relevant they actually are. Kyrpides and Ouzounis hypothesized that cognate protein–mRNA interactions may represent an ancient mechanism for autoregulation of mRNA stability (9,10), but structural and mechanistic aspects of their proposal have never been explored in detail. Altogether, the rapid growth of the number of experimentally verified mRNA-binding proteins, both cognate and non-cognate, has now created a strong incentive to search for the functional significance of such interactions and, even more fundamentally, the basic physico-chemical rules that guide them.

Related to this, we have recently shown that pyrimidine (PYR) density profiles of mRNA sequences tend to closely mirror sequence profiles of the respective cognate proteins capturing their amino-acid affinity for pyridines, chemicals closely related to PYR (15). These findings provided strong support for the stereo-chemical hypothesis concerning the origin of the genetic code, the idea that the specific pairing between individual amino acids and cognate codons stems from direct binding preferences of the two for each other (16–21). However, based on our results, such binding complementarity may exist predominantly at the level of longer polypeptide and mRNA stretches rather than individual amino acids and codons. Stimulated by these findings, we hypothesized that PYR-rich regions in mRNAs and protein stretches encoded by them may bind each other in a complementary fashion, a feature encoded directly in the universal genetic code (15). Although strongly suggestive, these findings remained silent about the potentially equivalent complementarity on the side of purines (PUR) as well as any details concerning specific nitrogenous bases. In addition to confirming our previous results using a completely orthogonal approach, the present study provides strong novel evidence along both of these two key lines.

## MATERIALS AND METHODS

### Analysis of contacts between amino-acid side chains and RNA nucleobases

All available structures of protein–RNA complexes (both X-ray and nuclear magnetic resonance structures) were downloaded from the Protein Data Bank (PDB) (22) in September 2012 using the 30% protein sequence identity and 3 Å resolution (for X-ray structures) cutoffs. The initial set was further manually filtered to exclude complexes containing double-stranded RNAs or mature transfer RNAs. The structures of the complete *Saccharomyces cerevisiae* (23), *Escherichia coli* (24) and *Thermus thermophilus* (25) ribosomes with the highest crystallographic resolution as well as the 50S subunit of the *Deinococcus radiodurans* (26) and *Haloarcula marismortui* ribosome were also included in the set. This resulted in a total of 299 individual PDB structures (Supplementary Table S1). An amino-acid residue and an RNA base were considered to be neighbors and form a contact if their centers of geometry were separated by less than a given cutoff distance. All the results reported in the main manuscript are given for the cutoff of 8 Å, whereas for testing purposes, this cutoff was also varied between 6 and 10 Å with a 0.25 Å step. We separately analyzed contact statistics for residues having at least one neighboring base (set ‘1+’ with a total of 25 820 unique contacts for 8 Å cutoff), at least two neighboring bases (set ‘2+’ with a total of 16 331 unique contacts for 8 Å cutoff) or include only the two closest neighboring bases (set ‘2’ with a total of unique 12 040 contacts for 8 Å cutoff).

### Calculations of amino-acid interaction preferences

Amino acid/nucleobase preferences 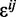 (with *i* = 1, … ,20 for amino acids and *j* = 1, … ,4 for bases) were estimated using the following standard distance-independent contact potential formalism with the quasi-chemical definition of the reference state (27–31):
(1)
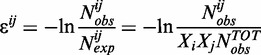

where 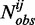 is the number of observed contacts between amino acid side chain of type *i* and nucleobase of type *j* in experimental structures, and 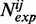 is the expected number of such contacts. The latter is calculated as the product of molar fractions of amino acid *i* and base *j* among all observed contacts (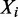 and 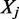, respectively) and the total number of all observed contacts 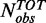.

Interaction preference scales of amino acids were obtained separately for guanine (‘G-preference’), adenine (‘A-preference’), cytosine (‘C-preference’), uracil (‘U-preference’), PUR (both G and A, ‘PUR-preference’) and PYR (both C and U ‘PYR-preferences’).

### Proteome data

The sequences of the complete human proteome (17 083 proteins) and coding sequences of their corresponding mRNAs were extracted from UniProtKB database (January 2013 release), with maximal-protein-evidence-level set at 4 (i.e. proteins annotated as ‘uncertain’ were excluded) and with only the reviewed Swiss-Prot (32) entries used for further analysis. The coding sequences of their corresponding mRNAs were extracted using the ‘Cross-references’ section of each of UniProtKB entry where out of several possible translated RNA sequences the first one satisfying the length criterion (RNA length = 3 × protein length + 3) was selected and its sequence downloaded from European Nucleotide Archive Database (http://www.ebi.ac.uk/ena). The protein as well as RNA sequences with only canonical amino acids or nucleotides were chosen for analysis. The complete set of mRNA/protein sequences used herein is included in the Supplementary Data. The average content of codons when it comes to individual nucleobases or PYR or PURs for all 20 amino acids (‘codon content’ scales) was extracted from the thus-obtained cognate mRNA and protein sequences.

### Correlation calculations

Pearson correlation coefficients (*R*) were calculated between nucleobase preferences and ‘codon content’ scales and between sequence profiles of nucleobase content for mRNAs and of different amino-acid preference scales for proteins from the complete human proteome set. Before comparison, the profiles were smoothed using a sliding-window averaging procedure; the window size of 21 residues/codons was used for all calculations.

### Analysis of statistical significance

Statistical significance (*P*-values) of the observed correlations was estimated using a randomization procedure involving random shuffling of the interaction preference scales. Each scale was shuffled one million times, and Pearson correlation coefficients (*R*) against codon content scales as well as for mRNA/protein profiles were calculated for each shuffled scale. The reported *P*-values correspond to the fraction of shuffled scales, which exhibit a higher absolute *R* than the original (|*R*| > |*R*_original_|) in the case of codon content comparisons, or for which <*R*> is higher in absolute value than <*R*_original_> in the case of sequence-profile comparisons.

The typical randomized scales whose distributions of correlation coefficients are depicted in the manuscript were chosen to be those, whose mean and standard deviation are the same as the average mean and the average standard deviation over all 10^6^ randomized scales in each case.

### Analysis of protein disorder and gene ontology (GO) classification

The average disorder for each protein sequence in the human proteome was predicted using IUpred server (33). Fourteen subsets of proteins displaying best or worst matching between their interaction preference profiles and nucleobase density profiles of their cognate mRNAs in term of Pearson *R* were extracted from the human proteome (top and bottom 10% cohorts) for the six cases of direct correspondence between nucleobase preferences and nucleobase composition profiles (e.g. protein G-preference versus G mRNA content, **G**_protein_-**G**_mRNA_, etc.) and for the G-preference versus PUR mRNA content one (**G**_protein_-**PUR**_mRNA_). Each of these subsets contains 1707 proteins, for which average disorder values were assigned. Means and standard deviations of the 14 thus-obtained distributions of average predicted disorder were compared with those of the entire human proteome (background). The significance of the mean difference from the background was estimated for each of the analyzed subsets using the Wilcoxon signed-rank test. The gene ontology (GO) analysis was performed for the same seven top 10% best-matching protein subsets using DAVID functional annotation server (34). The entire human proteome was used as background, and only the most significantly enriched functional terms with a DAVID EASE score (*P*-values) ≤10^−^^10^ were considered.

### Data visualization

The 3D structures of protein–RNA and amino acid/nucleobase complexes were visualized using PyMol (http://www.pymol.org/) (35). Contact statistics heat-map was produced using MATLAB (R2009a). Pearson *R* distributions for mRNA/protein profiles were processed and visualized using *Grace* (http://plasma-gate.weizmann.ac.il/Grace/).

## RESULTS

### Derivation of amino acid/nucleobase interaction preferences

**Figure 1.**
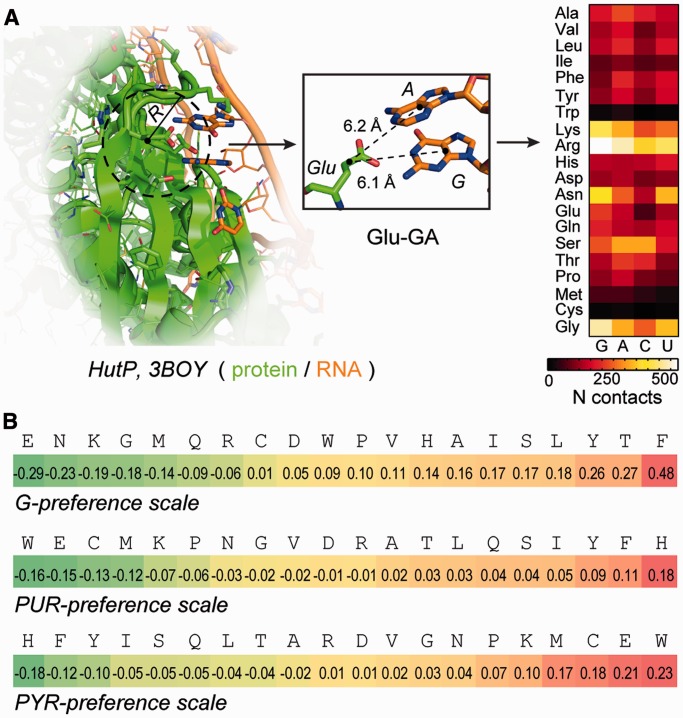
Derivation of amino acid/nucleobase interaction preference scales from known structures of RNA/protein complexes. (**A**) We define amino-acid side chains and RNA bases in a given complex to be contacting neighbors if their centers of geometry are less than a given cutoff radius *R* apart (*left* and *middle*) and merge contact statistics over the entire set of studied structures (*right*, ‘2+’ set with applied 8 Å cutoff). (**B**) Interaction preference scales of amino acids (in arbitrary units) for binding to guanines (G), PYR and PUR obtained from set ‘2+’ statistics using 8 Å cutoff (*panel A, right*). The scales are statistical analogs of relative free energy of binding (see ‘Materials and Methods’ section) with the prominently negative values corresponding to amino acid side chains having the highest affinities for bases of a given type and vice versa.

How differentiated and context-dependent are the preferences of amino acids to interact with specific nitrogenous bases? To address this question, we analyze contact interfaces of ∼300 high-resolution structures of different protein–RNA complexes including five ribosomal structures (Supplementary Table S1). We use distances between centers of geometry of amino-acid side chains and nucleotide nitrogenous bases in combination with a fixed cutoff to define contacting neighbors (Figure 1A). In this way, we isolate sequence-specific protein–RNA contacts (36–38) while ignoring non-specific interactions defined exclusively by protein or RNA backbones. We first present results for the distance cutoff of 8 Å following Shakhnovich *et al.* who established cutoffs between 7 and 8 Å to be optimal for residue-based statistical potentials describing protein–DNA interactions, albeit with a slightly different definition of reference points (28). However, all of our principal findings hold qualitatively for cutoffs between ∼6 and 9 Å as discussed later in the text. Finally, to differentiate cases in which an amino acid interacts with a single base only from denser, potentially more stereospecific contacts with more than one neighboring base within the cutoff, we separately merge contact statistics over the whole set of studied structures for amino acids having at least one neighboring base (set ‘1+’) or at least two neighboring bases (set ‘2+’, Figure 1A) within the cutoff.

Using standard distance-independent contact potential formalism (27–31), we subsequently derive scales of amino acid/nucleobase interaction preferences (Figure 1B and Supplementary Table S2) and use them to address the following questions: (i) how does the average composition of mRNA codons coding for a given amino acid relate to the preferences of this amino acid to interact with different nucleobases at protein/RNA interfaces and (ii) how does sequence density of different bases in mRNA-coding sequences relate to sequence profiles of amino-acid interaction preferences for these and other bases in cognate protein sequences?

### Amino acid interaction preferences and their codon content

**Figure 2.**
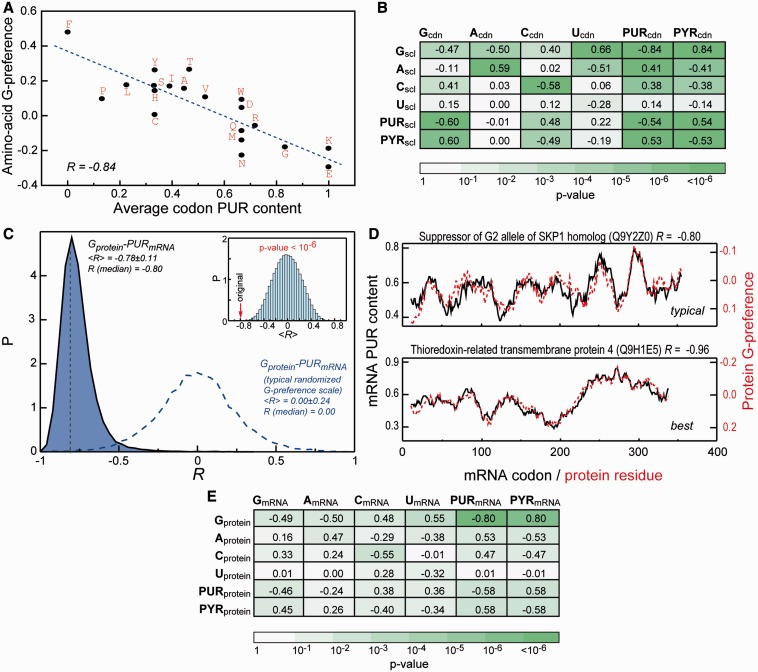
Relationship between nucleobase-binding preferences of amino acids and mRNA content at multiple levels. (**A**) Correlation between G interaction preferences of amino acids (Figure 1B) and the average PUR content of their codons in mRNAs of the entire human proteome. (**B**) Pairwise Pearson correlation coefficients (*R*) between base-binding preference scales of amino acids (‘scl’) and average base content of their codons (‘cdn’). (**C**) Distributions of correlation coefficients (*R*) between window-averaged PUR-content profiles of individual mRNA coding sequences and window-averaged G-preference sequence profiles of the respective proteins for the entire human proteome (window-size = 21). The dashed curve depicts the distribution of correlation coefficients calculated for a typical randomized G-preference scale. *Inset:* the distribution of the means of sequence-profile correlation coefficients for the human proteome (<*R*>) calculated for 10^6^ randomized G-preference scales. The *R* for the original G-preference scale is shown with an arrow. (**D**) Typical (*R = R*_median_) and best pairs of mRNA PUR-content (black curves) and protein G-preference profiles (red dashed curves) for human proteins. (**E**) Median pairwise Pearson correlation coefficients for comparison between nucleobase content profiles of mRNAs (subscript ‘mRNA’, *x*-axis) and base-preference-weighted protein sequence profiles (subscript ‘protein’, *y*-axis) over the entire human proteome. All results are based on the analysis of set ‘2+’ statistics. All data reported for preference scales are obtained using an 8 Å cutoff.

We first focus on contact statistics from set ‘2+’. Dinucleotides were found previously to exhibit potential for specific recognition of amino acids at protein–RNA interfaces (39) and have also been suggested as potential catalysts for amino acid synthesis in pre-biotic environments (40). Moreover, set ‘2+’ by definition also includes all instances where triplets of bases directly contact a given amino acid, which may be relevant in the context of the genetic code. Using set ‘2+’ statistics, we observe a remarkably strong correlation between preferences of amino acids to interact with guanine (G-preference, Figure 1B) and the average PUR content of their respective codons as derived from the complete human proteome with Pearson correlation coefficient *R* of −0.84 (Figure 2A). Negative Pearson correlation coefficients indicate matching between amino acid preferences and codon content owing to the way preference is defined (see ‘Materials and Methods’ section). Put differently, amino acids, which are predominantly encoded by PURs, display a strong tendency to co-localize with G at protein–RNA interfaces. This is also true, albeit at a somewhat weaker level of correlation, for matching between PUR composition of individual codons from the standard genetic table and the respective G-preferences if the statistics of codon usage in the human proteome is not included (*R* = −0.68, Supplementary Figure S1). The observed signal for G is statistically highly significant as evidenced by randomization calculations (*P*-value < 10^−^^6^, Figure 2B). Related to this, G-preference of amino acids inversely correlates with C and U content of their codons (Figure 2B). Somewhat less prominent, but still extremely significant correlations are observed for G- and C-preference of amino acids and the average G- and C-content of their codons (*R* of −0.47 and −0.58, respectively). On the other hand, the interface statistics for adenine (A) and uracil (U) do not correlate with their average usage in codons. In particular, the A-preference of amino acids correlates inversely with the A-content (*R* = 0.59) or directly with the U-content of their codons (*R* = −0.51), whereas the U-preference exhibits relatively low correlations throughout (Figure 2B). Finally, both PYR and PUR binding preferences of amino acids (Figure 1B) display significant correlations with PYR and PUR fraction in their codons with *R* of −0.54 and −0.53, respectively, and *P*-values < 10^−^^6^ in both cases. In other words, amino acids coded for by PYR-rich codons prefer to co-localize with PYR, and those coded for by PUR-rich codons with PUR at RNA–protein interfaces. Although similar in the present case, PYR- and PUR-preference scales need not necessarily be inverses of each other owing to the way preferences are defined, and we therefore here report and discuss both.

### Matching between sequence profiles of mRNAs and their cognate proteins

**Figure 3.**
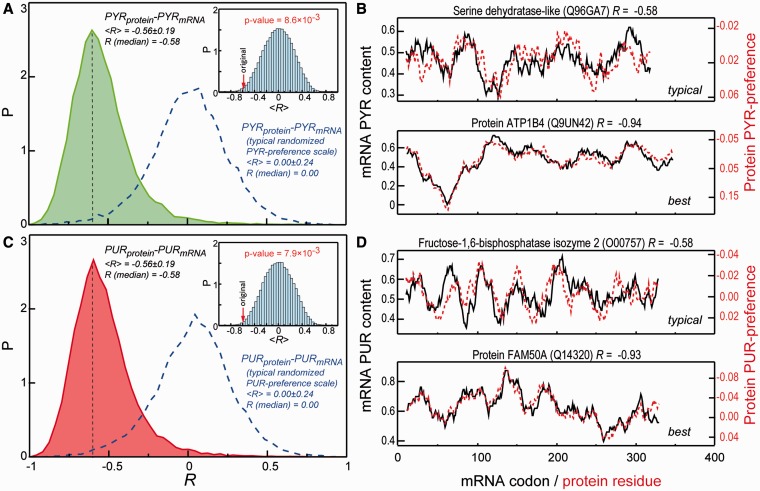
PYR/PUR mRNA sequence profiles strongly match PYR/PUR-preferences of cognate protein sequences. PYR (**A** and **B**) and PUR (**C** and **D**) amino-acid preference scales are given in Figure 1B. For details, please see the analogous captions to Figure 2C and D.

How do these observations translate if one compares complete mRNA-coding sequences with their cognate protein sequences? Owing to codon usage bias and non-uniform amino-acid composition of the human proteome, these results could in principle deviate significantly from the results obtained for individual codons and amino acids. To address this question, we calculate a Pearson *R* for every cognate mRNA/protein pair in the human proteome capturing the correlation between each mRNA sequence composition profile with the base-binding preference profile of its cognate protein sequence. Remarkably, we observe an extremely high level of matching between PUR density profiles of mRNAs and G-preference profiles of cognate protein sequences with a median Pearson *R* (*R*_median_) over the entire human proteome of −0.80 and a low *P*-value (<10^−^^6^) as determined by randomization (Figure 2C). In particular, the distribution of Pearson *R* values for this scale over the human proteome is significantly left shifted and shows only marginal overlap with the one calculated for a typical randomized interaction preference scale (Figure 2C). For illustration, we present sequence profiles for proteins of most abundant length (300–400 amino acids, Supplementary Figure S2) displaying typical (i.e. exhibiting a Pearson *R* equal to the population median) or best levels of correlation (Figure 2D). As is evident, the PUR density of mRNAs is quantitatively extremely well predicted by the G-binding preference profiles of cognate proteins even for typical human proteins (*R*_median_ = −0.80 and *P* < 10^−^^6^). We also observe significant matching between C-preference profiles for protein sequences and both C- and PYR-density profiles of their cognate mRNAs with *R*_median_ of −0.55 and −0.47, respectively (Figure 2E). In contrast, the A-preferences display significant matching with PYR-density profiles on the side of mRNA (Figure 2E; see also Supplementary Table S2 for the full report of profile correlations) with *R*_median_ of −0.53. Finally, strong and significant level of matching is observed for PYR-binding preferences of amino acids and PYR mRNA profiles as well as PUR-binding preferences of amino acids and PUR profiles (*R*_median_ of −0.58 in both cases and *P*-values of 8.6 × 10^−^^3^ and 7.9 × 10^−^^3^, respectively, Figure 3A and C). From the exemplary typical and best profiles (Figure 3B and D), it is clear that the PYR- and PUR-rich regions in mRNA code for stretches of amino acids in cognate proteins, which prefer to co-localize with PYR and PUR bases, respectively, at protein–RNA interfaces in the known 3D PDB structures. The typical level of similarity between sequence profiles is actually greater than what one might infer from *R*_median_ values, suggesting that Pearson correlation coefficient might not even be the optimal measure of deviation in this case. Importantly, this direct physico-chemical complementarity between mRNA and cognate protein sequences may be indicative of pronounced potential for complex formation between them, especially under circumstances when ‘peak’ regions become available for such interactions. Given the fact that a significant matching of profiles is detected at the level of primary sequences, we propose that the presence of extended unstructured protein and mRNA segments may be required for such binding. This suggestion agrees well with recent knowledge-based studies where RNA loops and bulges were found to be more likely to interact with amino-acid side chains in a specific manner (38,41).

**Figure 4.**
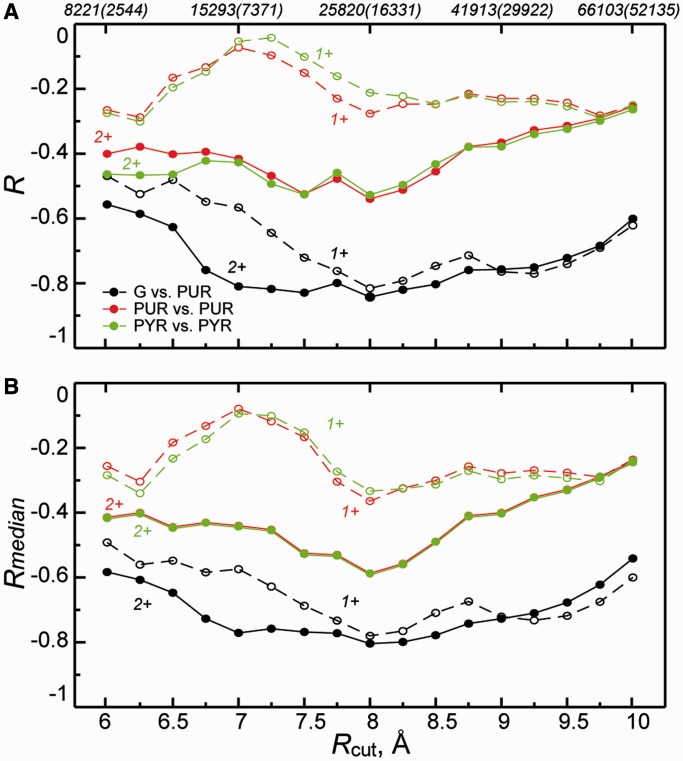
Effect of cutoff radius used to define protein–RNA contacts on observed correlations. (**A**) Dependence of Pearson correlation coefficients (*R*) between amino acid preference scales and average codon content on the cutoff radius for the two sets of statistics studied (‘1+’, ‘2+’). The total number of unique contacts in ‘1+’ and ‘2+’ (given in parentheses) sets obtained for each of used cutoff radii is indicated at the top of the panel. (**B**) Cutoff radius dependence of median pairwise Pearson correlation coefficients (*R*_median_) for comparison between nucleobase content profiles of mRNAs and base-preference-weighted protein sequence profiles over the entire human proteome (color code the same as in panel A).

**Figure 5.**
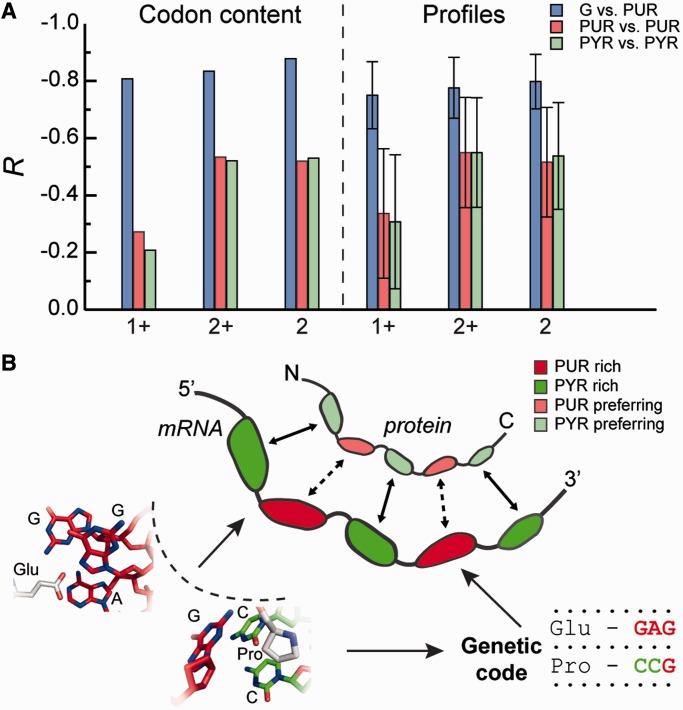
Physico-chemical origins of the mRNA/protein relationship. (**A**) Correlation coefficients (*R* and *<R>* with standard deviations) between PYR or PUR average codon content (‘Codon content’) and respective mRNA profiles (‘Profiles’) calculated for G- (blue), PUR- (red) and PYR- (green) binding preferences of amino acids, which were obtained using different amino acid neighbor statistics (1+, 2+ or 2). (**B**) A model of physico-chemical complementarity between proteins and cognate mRNAs. Preferential interactions of amino acids with PYR or PUR define their codon content in the genetic table and facilitate complementary interactions between PYR/PUR-rich mRNA regions and PYR/PUR preferring regions in proteins. The opposite behavior of adenines and guanines adds an additional layer of complexity in the case of PURs as signified by dashed arrows in the model. Note: polymer sizes not drawn to scale.

How sensitive is the level of matching to the choice of cutoff distance used to define contacting amino acids and nucleobases in protein/RNA complexes? To address this question, we have repeated the aforementioned analysis for a range of different cutoff values going from 6 to 10 Å in steps of 0.25 Å (Figure 4). Overall, for set ‘2+’, our findings are largely robust to the choice of the exact cutoff in this range, albeit with a somewhat lower level of significance for longer cutoffs. However, the majority of the signal is lost if one uses the ‘1+’ set, except for G-preference and PUR-content (Figure 5A) and A-preference and PYR-content (Supplementary Table S2). This observation strongly suggests that close dense packing of nucleobases around amino acids may be required for specificity in cognate complex formation. Although interfaces may be dynamic and liquid-like, as we have suggested before, they may still need to be densely packed. Interestingly, if one reduces the 2+ set by including only the two closest bases in contact with a given amino acid (set ‘2’), the signal for G-preference/PUR-content even further improves by several percentage points (Figure 5A), and the same holds for C-preference/C-content and A-preference/PYR-content (Supplementary Table S2).

To further study the role of protein structural disorder in matching, we have analyzed the levels of the predicted disorder of the top and the bottom 10% of proteins when it comes to the degree of mRNA/protein profile matching as captured by Pearson *R* coefficient (see ‘Materials and Methods’ section). We have done this for the six cases of direct comparison whereby the same base type is used for both protein preference and mRNA profile density (**G**_protein_-**G**_mRNA_, **A**_protein_-**A**_mRNA_, **C**_protein_-**C**_mRNA_, **U**_protein_-**U**_mRNA_, **PUR**_protein_-**PUR**_mRNA_ and **PYR**_protein_-**PYR**_mRNA_) and also for the case displaying the strongest signal in our analysis (**G**_protein_-**PUR**_mRNA_). Importantly, in the case of **G**_protein_-**G**_mRNA_, **A**_protein_-**A**_mRNA_ and **C**_protein_-**C**_mRNA_ matching, we do observe a pronounced tendency for the top and the bottom 10% cohorts to be significantly enriched (top 10%) and depleted (bottom 10%) in disordered proteins (Supplementary Table S3), whereas in the case of **U**_protein_-**U**_prof_ matching, the situation is reversed. Interestingly, for **PUR**_protein_-**PUR**_mRNA_, **PYR**_protein_-**PYR**_mRNA_ and **G**_protein_-**PUR**_mRNA_ matching, one observes slight disorder enrichment in both top and bottom cohorts. The most prominent shift of the distribution of predicted average disorder toward higher disorder as compared with background is observed for the top 10% cohort of proteins displaying strong matching between C-preference profiles of their sequences and the C-content of their cognate mRNAs (**C**_protein_-**C**_mRNA_, Supplementary Table S3, Supplementary Figure S3). One might argue that this effect could just be related to compositional properties of such protein and mRNA pairs, whereby disordered proteins are simply encoded by C-rich sequences. However, the differences between nucleobase compositions of mRNAs from the **C**_protein_-**C**_mRNA_ top 10% cohort and the complete proteome are minor, suggesting that the underlying explanation might be more complex (Supplementary Figure S3).

Which biological functions might be associated with a high level of complementarity between proteins and cognate mRNAs? To address this question, we have performed GO analysis for seven different top 10% subsets of proteins displaying strong matching with cognate mRNAs (see ‘Materials and Methods’ section for details). In Supplementary Table S4, we report the most significantly enriched biological functions (using a *P*-value cutoff of 10^−^^10^) shared by proteins from the analyzed cohorts. In a striking agreement with our hypothesis, in most cases, we observe pronounced enrichment of terms related to nucleic-acid/protein interactions, including regulation of RNA metabolic processes, ribonucleoprotein complexes and transcription. The latter, in particular, allows one to speculate that protein tendencies to associate with cognate mRNA might be used by the cells to modulate gene expression pathways. What is more, PUR or PYR density profiles of mRNAs are identical to PUR or PYR density profiles of coding-strand DNA sequences (with Us being replaced by Ts). Although based on our statistical potentials, we cannot say anything about T-binding preferences of amino acids, it is possible that our results may be generalizable even to DNA-protein interactions as well as other RNA-protein interactions. One should also mention that depending on the particular type of matching, other biological functions also tend to be enriched. For instance, the **U**_protein_-**U**_mRNA_ top 10% subset displays significant enrichment of membrane proteins, whereas **G**_protein_-**PUR**_mRNA_ top cohort seems to be populated by extracellular proteins and particularly those involved in the functioning of the innate immune system. Altogether, our preliminary GO analysis illustrates significant functional differences between proteins that strongly complement their cognate mRNAs and the rest of the human proteome, and these findings will be further explored in another manuscript.

## DISCUSSION

High levels of matching between base-binding-preference profiles of proteins and PYR- or PUR-density profiles of cognate mRNA-coding sequences, defined primarily by amino acid preferences to co-localize with G and C bases at RNA/protein interfaces, allow one to speculate that direct complementary binding interactions may be a key element underlying the whole mRNA/protein relationship when it comes to both its evolutionary development as well as present day biology (Figure 5B). This agrees well with and significantly extends our previous findings where we have shown that protein sequence profiles of amino acid affinity for PYR analogs (42–44) mirror PYR density profiles of cognate mRNA sequences (15). It should be emphasized, however, that our present results are based exclusively on the statistics of direct amino acid/nucleobase contacts at RNA/protein interfaces. It is therefore still possible that the driving force for interactions between mRNAs and cognate proteins is non-specific (e.g. binding of positively charged amino acid side chains to RNA phosphate groups), whereas complementary interactions actually confer specificity to binding.

Moreover, our results provide a clear evolutionary perspective concerning the physico-chemical origins of translation in line with the stereo-chemical hypothesis of the origin of the genetic code (16–21). In particular, our results give strong support to the possibility of direct templating of proteins from mRNAs in the era before the development of ribosomal decoding and code’s fixation in that era (17,45). In this framework, ancient amino acids associated with mRNA directly following their intrinsic physico-chemical preferences as outlined here. However, the fact that an analogous effect is not seen for all bases, especially adenine and uracil, supports the possibility that in addition to physico-chemical rationales in the context of direct binding other evolutionary forces were also responsible for shaping the genetic code as suggested before (19). Our results are most consistent with the possibility that the early stereo-chemical phase in code’s development was dominated by G- and C-rich codons, as strongest correlations are seen for precisely these bases. If the basic structure of the early genetic code was defined by such codons, but was later modulated by the inclusion of A and U bases, this might explain why G-affinity of amino acids in present-day protein sequences closely follows PUR density profiles in cognate mRNAs. Interestingly, Trifonov and coworkers have suggested that the first codons were G- and C-rich on the basis of a consensus analysis of 40 different criteria (46).

Importantly, it should be emphasized that the stereo-chemical hypothesis of the code’s origin may differ from the cognate mRNA/protein complementary interaction hypothesis in terms of its evolutionary underpinnings. Direct templating of proteins from mRNAs in ancient systems (the coding aspect of the stereo-chemical hypothesis) does not necessarily imply that modern proteins directly interact with their own mRNA (complementary interaction hypothesis). However, our findings support the possibility that the origin of the genetic code and potential complementarity between proteins and cognate mRNAs might have the same physico-chemical background. It is well possible that other independent influences have shaped both effects, and the two hypotheses leave ample room for such refinements. However, we would like to stress that in our view, the two hypotheses are inter-linked: cognate binding is on the one hand a reasonable consequence of the stereochemical hypothesis, but on the other hand, it also gives a potential biological rationale for the early development of the code to begin with, such as stabilization of RNA structures by bound polypeptides, as has been suggested before (45).

There are a number of open challenges concerning the aforementioned proposal. First and foremost, the structural features of mRNAs and cognate proteins impose severe constraints on any putative complementarity between the two. Namely, with the contour length of the mRNA coding part being ∼4.5 times longer than that of a cognate protein, it is not clear what structural arrangements may be consistent with any complementary interactions. We would like to suggest that structures of such complexes may be dynamic and liquid-like with mRNA stretches enveloping and solubilizing cognate protein stretches (15). Second, with many mRNAs and proteins being well-folded and compact for most of the time, it remains to be studied when and how opportunities could arise for the complementarity between their primary sequences to be of relevance. It is possible that, if at all realistic, such complementary binding might be functionally important precisely in those situations where both polymers are unstructured such as during translation, export and degradation, as a consequence of thermal stress or in the case of intrinsically unstructured proteins. However, we do not exclude the possibility of complementary interactions even in the folded state. Finally, concerning the origin of the genetic code, it is not clear how the final well-defined structure of the code could have arisen based on still partially non-specific large-scale binding interactions between mRNAs and cognate proteins. As suggested before, it is possible that the answer lies in a combination of different influences (19). Future research should shed light on these and related questions.

These challenges notwithstanding, our findings provide strong evidence that the ability to interact with mRNA might be a widespread phenomenon in the cell involving not only cognate proteins but also other proteins based on similar principles. The potential significance of such physico-chemical complementarity between mRNAs and proteins potentially extends to all facets of nucleic acid and protein biology in the modern cell including transcription/translation regulation (9,10,47,48), mRNA transport and localization (49,50), processing and decay (51), structure of ribonucleoproteins (52) and others (2–5,53,54). Our preliminary GO analysis has demonstrated a significant enrichment of functions related to association with nucleic acids for the subsets of proteins that complement their cognate mRNAs strongly, and these findings will be explored in more detail in future work.

## SUPPLEMENTARY DATA

Supplementary Data are available at NAR Online.

Supplementary Data
