# Supplementary material for: Evidence of direct complementary interactions between messenger RNAs and their cognate proteins
Source: Nucleic Acids Res. 2013 Jul 18;41(18):8434–43. doi: 10.1093/nar/gkt618 (PMC3794581; doi:10.1093/nar/gkt618)
Supplement: Supplementary Data [file supp_41_18_8434__index.html]

Evidence of direct complementary interactions between messenger RNAs and their cognate proteins — Supplementary Data 

# Evidence of direct complementary interactions between messenger RNAs and their cognate proteins

## Supplementary Data

files

**Files in this Data Supplement:**

- Supplementary Data - zip file
