# Supplementary material for: Evidence of direct complementary interactions between messenger RNAs and their cognate proteins
Source: Nucleic Acids Res. 2013 Jul 18;41(18):8434–43. doi: 10.1093/nar/gkt618 (PMC3794581; doi:10.1093/nar/gkt618)
Supplement: Supplementary Data [file supp_gkt618_suppl_data_updated.zip › PolyanskyAA_NAR_SI_revised_02072013.pdf]

Supplementary Data

Evidence of direct complementary interactions between messenger RNAs and their cognate proteins

Anton A. Polyansky and Bojan Zagrovic*

Department of Structural and Computational Biology, Max F. Perutz Laboratories, University of Vienna, Campus Vienna Biocenter 5, A-1030 Vienna, Austria

* To whom correspondence should be addressed. Tel: +43 1 4277 52271; Fax: +43 1 4277 9522; Email: bojan.zagrovic@univie.ac.at

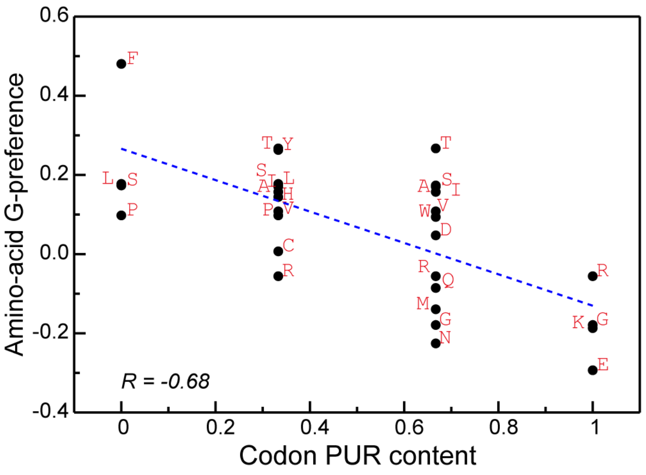

Figure S1. Correlation between G interaction preferences of amino acids and the PUR content of their individual codons.

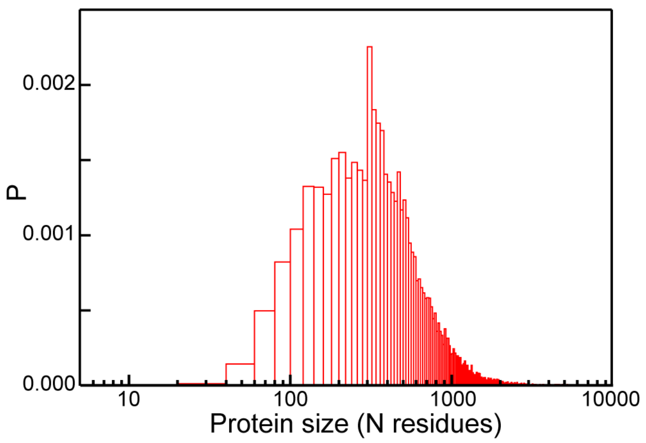

Figure S2. Normalized protein size distribution (plotted on a logarithmic scale) in the analyzed human proteome.

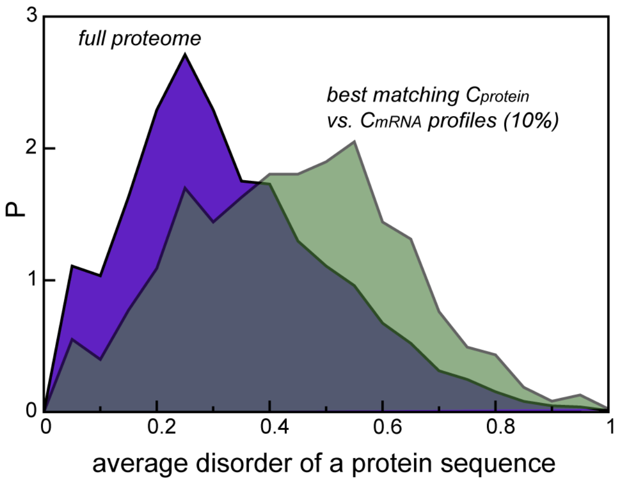

	%G	%A	%C	%U	%PUR	%PYR
full proteome	27.6	25.1	27.6	19.7	52.8	47.2
$C_{protein}$-C_{mRNA} (top 10%)	28.6	25.0	29.0	17.5	53.6	46.4

Figure S3. Distribution of the average disorder of protein sequences (calculated using IUPred server, see Method) from the entire human proteome (violet) and the top 10% of proteins displaying best matching between C-preference profiles and C density profiles of their cognate mRNAs (green). The average nucleotide composition (in percentage) of mRNAs from both analyzed sets are given in the table below.

Table S1. PDB structures used for the analysis of knowledge-based interaction preferences. Note that eukaryotic and bacterial ribosome structures were deposited in several separate PDB files.

Set	N	PDBs
**X-ray protein-RNA complexes**	237[a]	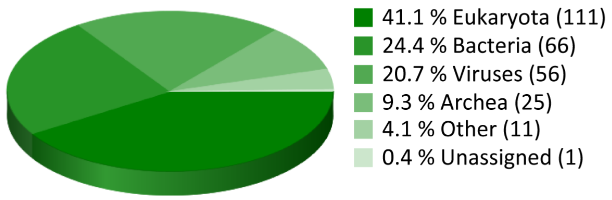
**NMR protein-RNA complexes**	52[b]	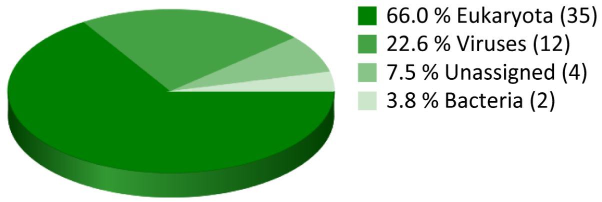
**Ribosomes**	10	
Saccharomyces cerevisiae	4	3U5B, 3U5C, 3U5D, 3U5E
Escherichia coli	2	3R80, 3R8T
Thermus thermophilus	2	3V2C, 3V2O
Deinococcus radiodurans (50S)	1	2ZJR
Haloarcula marismortui (50S)	1	3OW2

[a] PDB codes: 1A34, 1A9N, 1AQ3, 1AV6, 1BMV, 1C9S, 1CX0, 1DDL, 1FXL, 1G2E, 1GTF, 1GTN, 1H2C, 1HQ1, 1I5L, 1JBS, 1JID, 1K8W, 1KQ2, 1M5K, 1M5O, 1M8V, 1M8W, 1N1H, 1N35, 1N38, 1PGL, 1Q2R, 1R3E, 1RLG, 1S03, 1SDS, 1SJ3, 1SJ4, 1SJF, 1TFW, 1U0B, 1URN, 1UTD, 1UTF, 1UVI, 1UVJ, 1UVK, 1UVL, 1UVM, 1UVN, 1VBX, 1WPU, 1WSU, 1XOK, 1YTU, 1YTY, 1YVP, 1YYW, 1YZ9, 1ZBH, 1ZDH, 1ZDJ, 1ZE2, 1ZH5, 1ZHO, 1ZL3, 2A1R, 2AB4, 2ANN, 2ANR, 2ASB, 2ATW, 2BS1, 2BTE, 2BU1, 2BX2, 2DB3, 2E9T, 2E9Z, 2EC0, 2F8K, 2FZ2, 2G4B, 2GIC, 2GJW, 2GXB, 2HVY, 2HW8, 2HYI, 2I82, 2I91, 2IX1, 2J0S, 2JEA, 2JLU, 2NZ4, 2OIH, 2OZB, 2PJP, 2PO1, 2PXD, 2PY9, 2Q66, 2QUX, 2R7R, 2R8S, 2RFK, 2UWM, 2V3C, 2VNU, 2VOD, 2VOO, 2VPL, 2X1A, 2X1F, 2XD0, 2XDB, 2XGJ, 2XLK, 2XNR, 2XS2, 2XZO, 2Y8W, 2YJY, 2YKG, 3AEV, 3AF6, 3AGV, 3AHU, 3AVT, 3AVU, 3AVW, 3AVX, 3AVY, 3BOY, 3BSB, 3BSO, 3BSX, 3BT7, 3BX2, 3BX3, 3CUL, 3D2S, 3DD2, 3DH3, 3EGZ, 3EX7, 3FHT, 3FTE, 3G0H, 3G8T, 3G96, 3G9C, 3G9Y, 3GIB, 3GPQ, 3H5Y, 3HAX, 3HHN, 3HJW, 3HL2, 3HSB, 3I5X, 3IAB, 3ICE, 3IE1, 3IRW, 3K49, 3K5Q, 3K61, 3K62, 3K64, 3KLV, 3KMQ, 3KOA, 3L3C, 3LWV, 3M7N, 3MDI, 3MJ0, 3MOJ, 3MQK, 3MXH, 3NDB, 3NL0, 3NMR, 3NMU, 3NNC, 3NVI, 3O7V, 3O8C, 3OIN, 3OL6, 3P6Y, 3PEY, 3PF4, 3PF5, 3PTX, 3PU4, 3Q0Q, 3Q0R, 3Q0S, 3QG9, 3QGB, 3QGC, 3QJJ, 3QJL, 3QSU, 3R2C, 3R9W, 3RC8, 3RER, 3RW6, 3SIU, 3SN2, 3SNP, 3SQW, 3T3O, 3T5N, 3TMI, 3UCU, 3UD4, 3V6Y, 3V71, 3V74, 3V7E, 3VNV, 4AL5, 4AL6, 4ALP, 4AM3, 4DWA, 4ED5, 4EI1, 4F02, 4F3T, 4FTB, 4G0A, 5MSF, 6MSF, 7MSF

[b] PDB codes: 1A1T, 1AUD, 1BIV, 1DZ5, 1ETF, 1FNX, 1NYB, 1QFQ, 1T4L, 1U6P, 1ULL, 1WWD, 1WWE, 1WWF, 1WWG, 1ZBN, 2A9X, 2AD9, 2ADB, 2ADC, 2B6G, 2C06, 2CJK, 2ERR, 2HGH, 2I2Y, 2IHX, 2KDQ, 2KFY, 2KG0, 2KG1, 2KH9, 2KM8, 2KX5, 2KXN, 2L2K, 2L3C, 2L3J, 2L41, 2L5D, 2LA5, 2LBS, 2LEB, 2LEC, 2LI8, 2LUP, 2RQC, 2RRA, 2RS2, 2XC7, 2XFM, 2YH1

Table S2. Correlations between window-averaged profiles of base density of individual mRNA coding sequences and amino-acid interaction preferences for different bases of the respective protein sequences for the entire human proteome (*attached as Table_S2.xls file*).

Table S3. Average sequence disorder in 14 subsets of proteins displaying best (top 10%) and worst (bottom 10%) matching between their interaction preference profiles and nucleotide density profiles of their cognate mRNAs and the entire human proteome (*attached as Table_S3.xls file*).

Table S4. Results of Gene Ontology analysis for 7 subsets of proteins displaying best matching (top 10%) between their interaction preference profiles and nucleotide density profiles of their cognate mRNAs. Only the most significantly enriched functions (according to DAVID EASE score or adjusted p-values $\leq 10^{-10}$) are reported (*attached as Table_S4.xls file*).
